# Trace element supplementation in hemodialysis patients: a randomized controlled trial

**DOI:** 10.1186/s12882-015-0042-4

**Published:** 2015-04-11

**Authors:** Marcello Tonelli, Natasha Wiebe, Stephanie Thompson, David Kinniburgh, Scott W Klarenbach, Michael Walsh, Aminu K Bello, Labib Faruque, Catherine Field, Braden J Manns, Brenda R Hemmelgarn, for the Alberta Kidney Disease Network

**Affiliations:** Department of Medicine, University of Calgary, 3280 Hospital Dr NW, Calgary, AB T2N 4Z6 Canada; Department of Medicine, University of Alberta, Edmonton, Canada; Department of Physiology & Pharmacology, Calgary, Canada; Department of Medicine, McMaster University, Hamilton, Canada; Population Health Research Institute, Hamilton Health Sciences/McMaster University, Hamilton, Canada; Department of Clinical Epidemiology and Biostatistics, McMaster University, Hamilton, Canada; Department of Agricultural, Food & Nutritional Science, University of Alberta, Edmonton, Canada

**Keywords:** Hemodialysis, Selenium, Zinc

## Abstract

**Background:**

People with kidney failure are often deficient in zinc and selenium, but little is known about the optimal way to correct such deficiency.

**Methods:**

We did a double-blind randomized trial evaluating the effects of zinc (Zn), selenium (Se) and vitamin E added to the standard oral renal vitamin supplement (B and C vitamins) among hemodialysis patients in Alberta, Canada. We evaluated the effect of two daily doses of the new supplement (medium dose: 50 mg Zn, 75 mcg Se, 250 IU vitamin E; low dose: 25 mg Zn, 50 mcg Se, 250 IU vitamin E) compared to the standard supplement on blood concentrations of Se and Zn at 90 days (primary outcome) and 180 days (secondary outcome) as well as safety outcomes.

**Results:**

We enrolled 150 participants. The proportion of participants with low zinc status (blood level <815 ug/L) did not differ between the control group and the two intervention groups at 90 days (control 23.9% vs combined intervention groups 23.9%, P > 0.99) or 180 days (18.6% vs 28.2%, P = 0.24). The proportion with low selenium status (blood level <121 ug/L) was similar for controls and the combined intervention groups at 90 days (32.6 vs 19.6%, P = 0.09) and 180 days (34.9% vs 23.5%, P = 0.17). There were no significant differences in the risk of adverse events between the groups.

**Conclusions:**

Supplementation with low or medium doses of zinc and selenium did not correct low zinc or selenium status in hemodialysis patients. Future studies should consider higher doses of zinc (≥75 mg/d) and selenium (≥100 mcg/d) with the standard supplement.

**Trial registration:**

Registered with ClinicalTrials.gov (NCT01473914)

**Electronic supplementary material:**

The online version of this article (doi:10.1186/s12882-015-0042-4) contains supplementary material, which is available to authorized users.

## Background

People with severe kidney disease follow a restricted diet aimed at reducing intake of sodium, potassium and phosphate [1], which may lead to nutritional deficiency. Although the potential for malnutrition in people with kidney disease is well recognized, blood levels of most vitamins and trace elements are rarely measured. Instead, many North Americans with severe kidney disease are routinely prescribed a “renal vitamin” which contains a mixture of B and C vitamins.

Recent evidence indicates that people with kidney failure are often deficient in zinc and selenium [2]. Potential benefits of zinc supplementation relevant to dialysis patients include improved immune function, taste sensitivity (perhaps reducing dietary sodium intake), and appetite [3]. Potential benefits of selenium supplementation include reduced risk of vascular disease and infection [4]. Although vitamin E supplements reduced serious cardiovascular morbidity in a randomized controlled trial of people with kidney failure [5], this treatment is not routinely used in dialysis patients. Thus supplementation of zinc, selenium, and vitamin E may benefit patients with kidney failure. Since patients with kidney failure already take many medications, it is logical to combine any new nutritional supplements with the existing standard renal vitamin to reduce pill burden. However, zinc and selenium may be toxic in large doses or when accumulated over time. Therefore, the optimal dose of zinc and selenium is unknown for people with kidney failure, in whom renal excretion of these elements is impaired or absent.

We did a randomized trial in hemodialysis patients evaluating a novel nutritional supplement consisting of zinc, selenium and vitamin E added to the contents of the standard renal supplement (B and C vitamins). We compared two doses of the new supplement with the standard supplement with respect to serum concentrations in hemodialysis patients over 180 days of supplementation.

## Methods

### Participants

Participants were recruited from the Northern and Southern Alberta Renal Programs (from November 2012 to June 2013, from dialysis wards in Edmonton, Calgary and Red Deer, Alberta. Written informed consent was obtained. The Universities of Alberta and Calgary research ethics boards approved the study. This trial is reported according to the CONSORT guidelines [6].

Adults (≥18 y) stable on thrice weekly hemodialysis for 3 to 36 months receiving Replavite (WN Pharmaceuticals Ltd; Coquitlam, BC, Canada) or an equivalent renal vitamin at baseline were eligible for inclusion. Patients who were allergic to or intolerant of zinc, selenium, vitamin E, Replavite or corn starch were excluded from the trial. We excluded pregnant patients or patients planning a pregnancy, scheduled kidney transplantation or gastrointestinal surgery, anticipating a switch in dialysis modality, or estimated life expectancy <6 months. We also excluded patients with an ostomy/short gut syndrome, participants in another clinical trial, patients with head and neck cancer diagnosed in the past 5 years (given theoretical risks associated with selenium supplementation in this population), and those taking zinc, selenium, or vitamin E supplementation at baseline.

### Interventions

Participants were randomly assigned to 1 of 3 interventions: the standard renal vitamin formulation (biotin 300 mcg, folic acid 1 mg, niacinamide 20 mg, thiamine 1.5 mg, cyanocobalamin 6 mcg, riboflavin 1.7 mg, pyridoxine 10 mg, ascorbic acid 100 mg; control), the standard formulation compounded with vitamin E (250 IU) and low doses of zinc (25 mg) and selenium (50 mcg), or the standard formulation compounded with vitamin E (250 IU) and medium doses of zinc (50 mg) and selenium (75 mcg) (Additional file 1: Table S1). The appearance (size, colour, and capsule) and taste of the 3 vitamin compounds were exactly the same. Doses were selected to minimize the risk of toxicity in the setting of kidney failure and incorporated information on the maximum recommended dietary intake for healthy people (zinc: 40 mg/d [7]; selenium 400 mcg/d [8]) as well as prior studies in this population.

Zinc 25 mg and selenium 50 mcg daily (low dose formulation) are standard doses for supplementation in the general population, and similar or higher doses have been used in previous studies of hemodialysis patients without evidence of toxicity [9-11]. The higher doses of zinc 50 mg and selenium 75 mcg daily contained in the medium dose formulation may be more suitable for dialysis populations. We did not study high dose supplementation with zinc or selenium (doses ≥75 mg or ≥100 mcg respectively).

### Outcomes

Predialysis serum zinc and selenium concentrations were measured at 90 and 180 days (or at early withdrawal) following the baseline visit. The primary outcome was the proportion of participants in the combined medium and low dose groups who have low zinc status at 90 days (<815 ug/L; low zinc status) [12], compared to the standard vitamin group. The proportions of participants with low zinc status at 180 days, and with low selenium status (<121 ug/L) [12] at 90 and 180 days were also compared between groups as secondary outcomes.

Other secondary outcomes included serum levels of zinc and selenium, inter-dialytic weight gain and salt sensitivity (recognition and detection thresholds using SALSAVE [Advantech Toyo Co; Tokyo, Japan] test strips). We also collected data on serious adverse events (death, life-threatening illness, hospitalization, persistent and significant disability) and non-serious adverse events potentially related to the trial interventions (self-reported tremor, colour and texture of fingernails, frequent vomiting [>3 per week], severe neutropenia [WBC <3.5x10^9^/L], severe anemia [Hb <60 g/L].) Because high (usually 150–250 mg/d) doses of zinc can interfere with copper metabolism [13], we also compared the proportion of patients in each group with low copper status [<1061 ug/L] [12] at the end of the study.

### Laboratory methods

Zinc, selenium and copper were measured using the Agilent 8800 ICP mass spectrometer with helium and oxygen as the reaction gases. The calibration range for Cu and Zn is 0.1-100ug/L, while that for Se is 0.01-10 mcg/L. In each batch of samples, calibrators and 2 sources of CRMs (Certified Reference Materials) were run prior to sample injections. The CRMs used in this analysis were Seronorm Trace Metals Serum Control Level 1 and 2 and Clinchek Trace Metals Serum Control Level 1 and 2. The CRMs were re-injected after every 10 samples and the results were accepted within 20% range of the target values. Observed CV% (within run-between run) ranged from 4.3-9.2% and 1.2-8.3% for zinc and selenium respectively. For each sample batch, some samples were randomly picked as duplicates. The difference in percentage between duplicates was calculated and the results were accepted if the difference was less than 15%. If this criterion was not met, the sample was repeated in a different run.

### Covariates

We collected data on the following demographic variables: age, gender, and ethnicity (white or otherwise). Obesity (body mass index ≥30 kg/m^2^) and current smoking status were recorded as well as the following baseline comorbidities: cancer, coronary artery disease, diabetes, heart failure, hypertension, peripheral vascular disease, seizure disorder and stroke.

### Trial design

This was a randomized, double-blind, active-control, 3-group, parallel trial that was registered with ClinicalTrials.gov (NCT01473914). Participants were asked to take the intervention orally, once per day after dialysis or the same time of day as they would generally finish dialysis, for 180 days following the baseline visit. The intervention assignments were equally allocated between groups using randomly-generated permuted blocks of 6 and 9. The serially numbered identical bottles were pre-filled with the trial vitamin compounds using lists of intervention assignments, one generated for each renal program, and labelled with the trial name, the site name, and the site and participant number. The randomization lists were kept in a locked cabinet by the statistician who generated the lists. Participants, study coordinators and study investigators with the exception of the study statistician were kept unaware of the intervention assignments. Lab personnel who were involved in outcome ascertainment (serum concentrations) were also unaware of the therapy that participants had received.

Data were collected via participant interviews, chart reviews and clinical databases at baseline, 30, 90, 180 days (when the trial intervention was stopped), and 30 days following the discontinuation of the trial intervention. Medical history, medication use and demographics were ascertained at baseline. Pre-dialysis blood samples were taken by qualified dialysis unit personnel at day 90 and day 180 and stored in a −80°C freezer at the Canadian Biosample Repository. The dialysis prescription, pre- and post-dialysis blood pressure and weight data collection, the salt sensitivity testing, the trial intervention compliance checked, and the serious and non-serious adverse event reviews were completed at each interview. Adverse event reviews were also completed 30 days following the discontinuation of the trial intervention. All adverse events were reviewed by the site investigators within 24 hours.

A sample size of 150 participants (approximately 50 participants per group) was chosen to provide 80% power (with a 5% type 1 error rate and a 30% loss to follow-up) to detect a relative reduction in low zinc status of 33% (or an absolute reduction from 90% to 60%) between the combined medium and low dose groups and the standard vitamin group. No interim analyses were planned due to the short duration of the trial.

### Statistical analyses

All analyses were completed in Stata/MP 13.0 (www.stata.com). The primary analysis followed an intention-to-treat approach. In sensitivity analyses, missing outcome data were imputed using a last-value carried forward approach. Per protocol results were also generated. Baseline descriptive statistics were reported as counts and percentages, or medians and inter-quartile ranges, as appropriate.

For continuous outcome data, we used mixed regression models where participant was modelled as a random intercept, and intervention, time, the interaction between intervention and time, renal program (NARP, SARP) and baseline value were modelled as fixed effects. For inter-dialytic weight gain where there was a maximum of 9 time points (3 dialysis runs at 30, 90 and 180 days on intervention), residuals within the participant were modelled using an exponential covariance-variance matrix. The exponential covariance-variance matrix allows for non-equidistances between consecutive time points; the matrix has two parameters: a shared variance and a correlation which would be raised to the difference between any two time points. For dichotomous outcomes (e.g., low element status) we used simple *χ*^2^ tests. In sensitivity analyses, we used generalized linear mixed models (with a logistic link and the binomial family) and adjusted for intervention, time, their interaction, renal program and the baseline low element status. Means with 95% confidence intervals were reported, or counts and percentages, where appropriate. The means were adjusted for dialysis unit location and the relevant mean baseline value. P <0.05 was considered statistically significant.

## Results

### Participants

**Figure 1:**
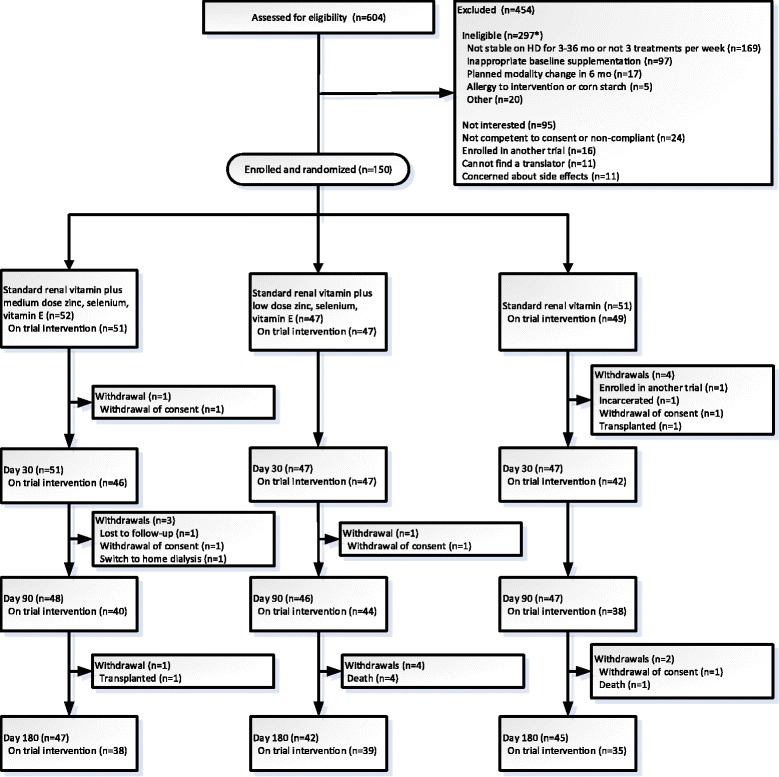
Participant flow diagram. All participants who received at least one dose of the trial intervention were included in the intention-to-treat primary analysis (51 in the medium dose group, 47 in the low dose group, and 49 in the standard dose group). *Participants may have more than one reason for ineligibility.

Six hundred and four hemodialysis patients were screened for inclusion in the trial; 454 were excluded (Figure 1). Characteristics of the 150 patients who were enrolled and randomized are shown in Table 1. Study flow is shown in Figure 1. The dataset was locked on March 21, 2014.

**Table 1:** **Demographics and clinical characteristics of participants**

	**Medium**	**Low**	**Standard**
	**dose**	**dose**	**dose**
N	52	47	51
Age, y	63 (59,67)	60 (57,64)	58 (54,62)
Male	39 (75)	35 (74.5)	37 (72.5)
Caucasian/white	41 (78.8)	36 (76.6)	42 (82.4)
BMI, kg/m^2^	29 (27,30)	31 (29,34)	27 (25,29)
Smoker	4 (7.8)	8 (17)	13 (25.5)
Primary cause of ESRD			
Diabetes	22 (42.3)	26 (55.3)	19 (37.3)
Glomerulonephritis	10 (19.2)	9 (19.1)	7 (13.7)
Hypertension	7 (13.5)	4 (8.5)	8 (15.7)
PCKD	1 (1.9)	1 (2.1)	6 (11.8)
Other	11 (21.2)	7 (14.9)	11 (21.6)
Comorbidities			
Cancer	8 (15.7)	8 (17)	7 (13.7)
CAD	21 (41.2)	16 (34)	15 (29.4)
Diabetes^1^	9 (17.6)	4 (8.5)	5 (9.8)
Heart failure	12 (23.5)	6 (12.8)	12 (23.5)
Hypertension	41 (80.4)	38 (80.9)	33 (64.7)
PVD	4 (7.8)	2 (4.3)	2 (3.9)
Seizure disorder	2 (3.9)	2 (4.3)	3 (5.9)
Stroke	3 (5.9)	5 (10.6)	3 (5.9)
Salt sensitivity			
No recognition	10 (19.6)	9 (19.2)	10 (19.6)
Recognition^2^	0.8 (0.7,0.9)	0.8 (0.7,0.8)	0.8 (0.7,0.8)
Dectection^2^	0.7 (0.7,0.8)	0.7 (0.6,0.7)	0.7 (0.6,0.7)
Serum concentrations, ug/L			
Copper	1131 (962,1266)	1066 (919,1237)	1024 (864,1213)
Selenium	139 (135,143)	137 (133,142)	135 (129,141)
Zinc	884 (851,917)	861 (823,898)	911 (867,955)
Low copper status	22 (42.3)	23 (48.9)	27 (52.9)
Low selenium status	8 (15.4)	9 (19.2)	14 (27.5)
Low zinc status	20 (38.5)	20 (42.6)	18 (35.3)

N (%) or mean (95% confidence intervals) where appropriate. Low copper status <1061 ug/L. Low selenium status <121 ug/L. Low zinc status <815 ug/L.

BMI body mass index, ESRD end-stage renal disease, PCKD poly cystic kidney disease, CAD coronary artery disease, PVD peripheral vascular disease.

^1^Those participants with diabetic nephropathy were not included in the counts for comorbid diabetes.

^2^In those participant with recognition of a salty taste.

The median age of participants was 62 years; 74% were male, and most were white (79%). Median BMI was 27 kg/m^2^ and 45% had diabetic nephropathy and a further 12% had co-morbid diabetes. Thirty-seven percent had low zinc status at baseline. The demographic and clinical characteristics of the treatment groups were generally comparable at baseline, although more control participants had low serum copper (53% vs 42% or 49%) and low selenium status (28% vs 15% or 19%). Overall adherence (assessed by counting the number of remaining capsules) was 83%.

### Zinc

**Figure 2:**
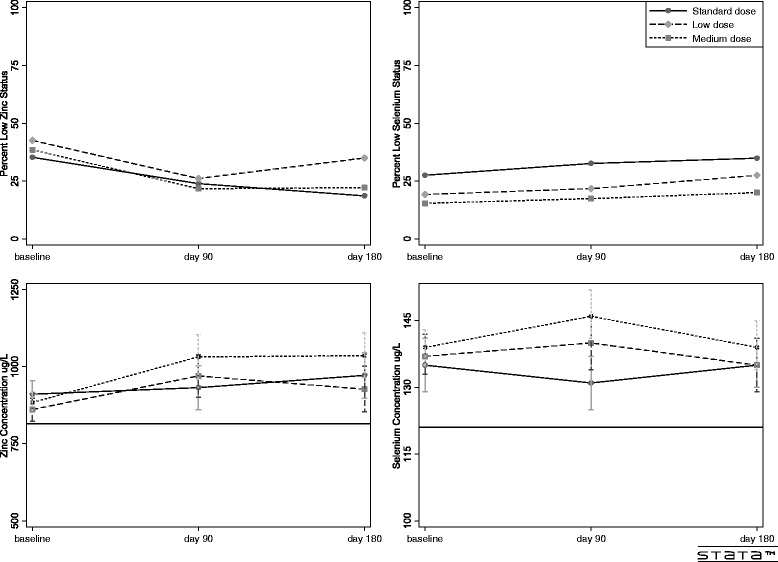
Zinc and selenium by timepoint. The left panels show the proportion of participants with low zinc status in the upper left panel and the mean serum zinc concentration of the participants in the lower left panel. The right panels show the proportion of participants with low selenium status in the upper right panel and the mean serum selenium concentration of the participants in the lower right panel. The lower panels have black horizontal lines depicting the thresholds of low element status (serum zinc <815 ug/L and serum selenium <121 ug/L). The solid line represents the control group. The dashed line represents the low dose intervention group and the short-dashed line represents the medium dose intervention group.

The proportion of participants with low zinc status declined during follow-up for all three treatment groups (Figure 2). The proportion of participants with low zinc status did not differ between the control group and the two intervention groups combined at 90 days (23.9% vs 23.9%, P > 0.99) or 180 days (control 18.6% vs combined intervention groups 28.2%, P = 0.24). Results were similar after adjustment for low zinc status at baseline (P = 0.91, P = 0.51). Mean serum zinc concentration also did not significantly differ between the control group and the combined intervention groups at 90 or 180 days (932 vs 998 ug/L, P = 0.11; 972 vs 982 ug/L, P = 0.23, respectively). However, serum zinc level was significantly higher in participants receiving the medium dose intervention (50 mg/d) vs control at 90 and 180 days (1032 vs 932 ug/L, P = 0.04; 1036 vs 972 ug/L, P = 0.04). Results were similar for the per-protocol analysis and the sensitivity analysis using imputed values (data not shown).

### Selenium

The proportion of participants with low selenium status was similar for controls and the combined intervention groups at 90 days (32.6 vs 19.6%, P = 0.09) and 180 days (34.9% vs 23.5%, P = 0.17). Results were similar after adjustment for low selenium status at baseline (P = 0.28, P = 0.67). As compared to controls, mean serum concentration was significantly higher in the combined intervention group at 90 days but not 180 days (143 vs 131 ug/L, P < 0.001; 137 vs 135 ug/L, P = 0.07). Mean serum selenium concentration was significantly higher in participants receiving the medium dose intervention (75 mcg/d) at 90 days (146 vs 131 ug/L, P < 0.001) and at 180 days (139 vs 135 ug/L, P = 0.03). Results were again similar for the per-protocol analysis and the sensitivity analysis using imputed values (data not shown).

### Salt sensitivity

Compared with controls, the recognition and detection of salt sensitivity was not significantly different for the combined intervention groups at day 90 or day 180 (recognition 32.6 vs 40.2%, and sensitivity 25.0 vs 26.1% at day 90; recognition 34.1 vs 42.7%, and sensitivity 28.1 vs 26.1% at day 180). Results were significant when controls were compared with the medium dose treatment group for recognition at day 90 (32.6 vs 55.3%, P = 0.03) but did not remain significant at day 180 (34.1 vs 46.8%, P = 0.22).

### Interdialytic weight gain

The weight gain between dialysis treatments was measured on nine occasions: all three runs during the weeks of day 30, day 90, and day 180. Mean weight gain was approximately 2 kg between each timepoint and for each group (Table 2). There were no significant differences between groups.

**Table 2:** **Outcomes at 90 and 180 days**

	**Medium dose**	**Low dose**	**Medium and low dose combined**	**Standard dose**	**P-value**
**At day 90**					
Zinc					
Low status, %	10 (21.7)	12 (26.1)	22 (23.9)	11 (23.9)	<0.99
Concentration, ug/L	**1032 (960,1104)**	970 (900,1039)	998 (945,1052)	932 (860,1003)	0.11
Selenium					
Low status, %	8 (17.4)	10 (21.7)	18 (19.6)	15 (32.6)	0.09
Concentration, ug/L	**146 (141,152)**	**140 (134,146)**	**143 (139,147)**	131 (125,137)	<0.001
Inter-dialytic weight gain, kg	1.96 (1.70,2.22)	2.20 (1.94,2.46)	2.08 (1.88,2.28)	2.01 (1.74,2.28)	0.64
Salt sensitivity					
Recognition improved	**26 (55.3)**	11 (24.4)	37 (40.2)	15 (32.6)	0.39
Sensitivity improved	11 (32.4)	7 (20.0)	18 (26.1)	8 (25.0)	0.91
**At day 180**					
Zinc					
Low status, %	10 (22.2)	14 (35.0)	24 (28.2)	8 (18.6)	0.24
Concentration, ug/L	**1036 (964,1109)**	927 (854,1001)	982 (928,1037)	972 (898,1046)	0.23
Selenium					
Low status, %	9 (20.0)	11 (27.5)	20 (23.5)	15 (34.9)	0.17
Concentration, ug/L	**139 (134,145)**	135 (129,141)	137 (133,142)	135 (130,141)	0.07
Inter-dialytic weight gain, kg	1.99 (1.69,2.29)	2.16 (1.85,2.47)	2.17 (1.95,2.40)	1.97 (1.66,2.28)	0.26
Salt sensitivity					
Recognition improved	22 (46.8)	16 (38.1)	38 (42.7)	15 (34.1)	0.24
Sensitivity improved	11 (31.4)	7 (20.6)	18 (26.1)	9 (28.1)	0.83

Count (percentage) and adjusted mean (with 95% confidence intervals) where appropriate; values were adjusted for the NARP site and the mean baseline value. P-values statistically compare the combined dose group to the standard group. Bolded values are significantly different from the standard group. Low selenium status <121 ug/L. Low zinc status <815 ug/L.

### Adverse events

There were no significant differences in the risk of serious and non-serious adverse events between groups (Table 3). There were a total of six deaths during the trial; none in the medium dose intervention group, 4 in the low dose intervention group and 2 in controls (P for controls vs treatment groups combined P = 0.65). There were a total of 29 hospitalizations and the proportion of participants hospitalized at least once were not significantly different between control and treatment recipients (P for controls vs treatment groups combined 0.79, 13.7 vs 17.2%)

**Table 3:** **Adverse events**

	**Medium dose**	**Low dose**	**Medium and low dose combined**	**Standard dose**	**Exact P-value**
Non-serious events	7 (13.7)	3 (6.4)	10 (10.2)	7 (14.3)	0.59
Self-reported tremor	0 (0)	1 (2.1)	1 (1.0)	0 (0)	<0.99
Color and texture of fingernails	3 (5.9)	1 (2.1)	4 (4.1)	2 (4.1)	<0.99
Frequent vomiting	0 (0)	1 (2.1)	1 (1.0)	2 (4.1)	0.26
Severe neutropenia	3 (5.9)	0 (0)	3 (3.1)	3 (6.1)	0.40
Severe anemia	1 (2.0)	0 (0)	1 (1.0)	0 (0)	<0.99
Low copper status	23 (50.0)	22 (47.8)	45 (48.9)	27 (58.7)	0.28
Serious events					
Death	0 (0)	4 (8.5)	4 (4.1)	2 (3.9)	0.65
Hospitalization^1^	8 (15.4)	9 (19.2)	17 (17.2)	7 (13.7)	0.79
Hospitalizations	9	12	21	8	

Counts (percentages) are reported. P-values statistically compare the combined dose group to the standard group. Bolded values are significantly different from the standard group. Low copper status <1061 ug/L.

^1^5 participants (3 in the low dose group and 1 in the standard dose group and 1 in the medium dose group) had 2 SAE-specific hospitalizations. In the above analysis, only 1 hospitalization is counted per participant (rather than per SAE-specific event).

A total of 17 participants reported non-serious adverse events such as tremor, changes in the color or texture of fingernails, vomiting, mild neutropenia, or mild anemia. The proportion of participants experiencing at least one of these events was not significantly different between control and treatment recipients (14.3 and 10.2%, respectively; p = 0.59).

## Discussion

Overall, supplementation with zinc and selenium had only modest effects on serum selenium levels and did not decrease the proportion of participants with low levels of either trace element. Results with the medium dose supplement (50 mg of zinc and 75 mcg of selenium) increased serum levels of zinc and selenium. Supplementation with zinc and selenium did not enhance salt recognition or sensitivity, although there were non-significant trends to improvement when the medium dose supplement was considered. There was no evidence that supplementation increased the risk of adverse effects.

Most previous studies of zinc supplementation in hemodialysis patients predominantly used doses of 50 mg/d, which safely increased zinc levels and (in one study) salt sensitivity [9,10,14]. Other studies used higher doses, zinc 100 mg/d, apparently without adverse effects [15,16]. There are fewer studies of selenium supplementation in dialysis populations, but 200 and 300 mcg/d were both used in short-term studies of hemodialysis patients, without an increased risk of adverse events [11,17,18]. We chose relatively low doses of zinc and selenium because we were concerned about the risk of toxicity. However, our results suggest that higher doses of oral supplementation may be required to correct low zinc and selenium status in hemodialysis patients.

Because of a previous RCT [5] suggesting that vitamin E supplementation prevented cardiovascular events in hemodialysis patients, we included 250 IU of tocopherol in the two active treatments. We would have preferred to use a higher dose of vitamin E, but this was not possible without an unacceptable increase in capsule size. Unfortunately, we cannot determine whether the lack of benefit of study drug on cardiovascular outcomes was due to low statistical power or an inadequate dose of tocopherol -- or because vitamin E supplementation does not prevent cardiovascular events in hemodialysis populations.

Why did we not observe improved serum concentrations of zinc or selenium? First, hemodialysis might remove unbound trace elements such as zinc and selenium from blood, meaning that higher maintenance doses would be required. Second, gastrointestinal absorption might be compromised in the presence of kidney failure, especially in the setting of nausea and vomiting (which is more common in dialysis patients than in the general population) [19]. Third, the severe dietary restrictions and frequent anorexia associated with kidney failure might lead to very low dietary trace element intake – meaning that unusually high doses are required to overcome deficiency. Fourth, although there is no *a priori* reason that co-administration with vitamins B, C and E should reduce absorption, it is possible that the combination therapy we studied somehow reduced bioavailability of zinc and selenium compared to monotherapy. Fifth, failure to take the supplements as requested is a possible explanation for the lower-than-expected effects on zinc and selenium status. Adherence in our study was 83% as assessed by capsule count but true adherence may have been lower. Finally, our study was powered primarily to detect an effect of treatment for the two supplement groups combined as compared with control. Our results are compatible with a small but still potentially beneficial effect of supplementation with medium dose zinc and selenium on serum levels – and suggest that higher doses warrant future study.

Our study has important strengths that should be considered when interpreting its results. First, it was a randomized, double-blind study that was done with minimal risk of bias. Second, all serum trace element assays were done using batched samples (to avoid assay drift) at a single reference laboratory. The laboratory followed rigorous quality assurance protocols reducing the potential for measurement error. However, our study also has some limitations. First, it was relatively small (N = 150) and used two active treatment arms, which likely reduced statistical power for comparisons of treatment vs placebo. Second, although it is one of the longest studies of trace element supplementation ever done in a kidney failure population, a longer study would be required to conclusively assess safety. In addition to reducing the frequency of low zinc and/or selenium levels, a future study demonstrating benefits for patient-important outcomes would be required to inform clinical practice. Third, assessment of zinc and selenium status is complex, and potentially affected by nutritional status as well as shifts between intracellular and extracellular compartments [20-22]. Although blood levels are considered acceptable measures of zinc and selenium status, the precise level that represents biological deficiency is not known for the general population or for dialysis patients. We did not evaluate biomarkers of zinc status such as serum metallothionein activity or plasma levels of glutathione peroxidase. Fourth, and most important, the doses of supplementation used were relatively conservative, which may have reduced the likelihood of showing a beneficial effect on trace element status in this population.

## Conclusions

In conclusion, we found no convincing evidence that supplementation with low or medium doses of zinc and selenium corrected abnormal trace element status in hemodialysis patients. Future studies of oral trace element supplementation should compare higher doses of zinc (≥75 mg/d) and higher doses of selenium (≥100 mcg/d) with control. If these higher doses are also insufficient to correct low blood levels of zinc and selenium, consideration of parenteral supplementation may be worthwhile.

## Additional file

Additional file 1: Table S1. Trial intervention formulations.

## References

[1] National Kidney Foundation: Nutrition and hemodialysis. In*.* New York: National Kidney Foundation, Inc, NY; 2013. https://www.kidney.org/sites/default/files/11-50-0136_nutri_hemo.pdf.

[2] Tonelli M, Wiebe N, Hemmelgarn B, Klarenbach S, Field C, Manns B (2009). Trace elements in hemodialysis patients: a systematic review and meta-analysis. BMC Med.

[3] Rucker D, Thadhani R, Tonelli M (2010). Trace element status in hemodialysis patients. Semin Dial.

[4] Rayman MP (2000). The importance of selenium to human health. Lancet.

[5] Boaz M, Smetana S, Weinstein T, Matas Z, Gafter U, Iaina A (2000). Secondary prevention with antioxidants of cardiovascular disease in endstage renal disease (SPACE): randomised placebo-controlled trial. Lancet.

[6] Schulz KF, Altman DG, Moher D, Group C (2010). CONSORT 2010 Statement: updated guidelines for reporting parallel group randomised trials. BMC Med.

[7] Food Sources of Zinc [http://www.dietitians.ca/Nutrition-Resources-A-Z/Factsheets/Minerals/Food-Sources-of-Zinc.aspx]

[8] Food Sources of Selenium [http://www.dietitians.ca/Nutrition-Resources-A-Z/Factsheets/Minerals/Food-Sources-of-Selenium.aspx]

[9] Mahajan SK, Prasad AS, Lambujon J, Abbasi AA, Briggs WA, McDonald FD (1980). Improvement of uremic hypogeusia by zinc: a double-blind study. Am J Clin Nutr.

[10] Rashidi AA, Salehi M, Piroozmand A, Sagheb MM (2009). Effects of zinc supplementation on serum zinc and C-reactive protein concentrations in hemodialysis patients. J Ren Nutr.

[11] Salehi M, Sohrabi Z, Ekramzadeh M, Fallahzadeh MK, Ayatollahi M, Geramizadeh B (2013). Selenium supplementation improves the nutritional status of hemodialysis patients: a randomized, double-blind, placebo-controlled trial. Nephrol Dial Transplant.

[12] LeBlanc A, Lapointe S, Beaudet A, Côté I, Dumas P, Labrecque F (2003). Étude sur l'Éstablissement de Valeurs de Référence d'Élément Traces et de Métaux dans le Sang, le Sérum et l'Urine de la Population de la Grande Région de Québec.

[13] Willis MS, Monaghan SA, Miller ML, McKenna RW, Perkins WD, Levinson BS (2005). Zinc-induced copper deficiency: a report of three cases initially recognized on bone marrow examination. Am J Clin Pathol.

[14] Jalali GR, Roozbeh J, Mohammadzadeh A, Sharifian M, Sagheb MM, Hamidian Jahromi A (2010). Impact of oral zinc therapy on the level of sex hormones in male patients on hemodialysis. Ren Fail.

[15] Mazani M, Argani H, Rashtchizadeh N, Ghorbanihaghjo A, Hamdi A, Estiar MA (2013). Effects of zinc supplementation on antioxidant status and lipid peroxidation in hemodialysis patients. J Ren Nutr.

[16] Rahimi-Ardabili B, Argani H, Ghorbanihaghjo A, Rashtchizadeh N, Naghavi-Behzad M, Ghorashi S (2012). Paraoxonase enzyme activity is enhanced by zinc supplementation in hemodialysis patients. Ren Fail.

[17] Adamowicz A, Trafikowska U, Trafikowska A, Zachara B, Manitius J (2002). Effect of erythropoietin therapy and selenium supplementation on selected antioxidant parameters in blood of uremic patients on long-term hemodialysis. Med Sci Monit.

[18] Zachara BA, Gromadzinska J, Zbrog Z, Swiech R, Wasowicz W, Twardowska E (2009). Selenium supplementation to chronic kidney disease patients on hemodialysis does not induce the synthesis of plasma glutathione peroxidase. Acta Biochim Pol.

[19] Davison SN, Jhangri GS, Johnson JA (2006). Cross-sectional validity of a modified Edmonton symptom assessment system in dialysis patients: a simple assessment of symptom burden. Kidney Int.

[20] Ashton K, Hooper L, Harvey LJ, Hurst R, Casgrain A, Fairweather-Tait SJ (2009). Methods of assessment of selenium status in humans: a systematic review. Am J Clin Nutr.

[21] Fairweather-Tait SJ, Collings R, Hurst R (2010). Selenium bioavailability: current knowledge and future research requirements. Am J Clin Nutr.

[22] Lowe NM, Fekete K, Decsi T (2009). Methods of assessment of zinc status in humans: a systematic review. Am J Clin Nutr.

