# Supplementary material for: Trace element supplementation in hemodialysis patients: a randomized controlled trial
Source: BMC Nephrol. 2015 Apr 11;16:52. doi: 10.1186/s12882-015-0042-4 (PMC4409771; doi:10.1186/s12882-015-0042-4)
Supplement: Additional file 1: Table S1. — Trial intervention formulations. [file 12882_2015_42_MOESM1_ESM.doc]

**Additional files**

Web Appendix Table 1. Trial intervention formulations

|  | **Medium dose** | **Low dose** | **Standard dose** |
| --- | --- | --- | --- |
| Zinc (as zinc sulfate), mg | 50 | 25 | 0 |
| Selenium (as sodium selenite), mcg | 75 | 50 | 0 |
| Vitamin E (D-alpha-tocopherol) (as succinate), IU | 250 | 250 | 0 |
| Biotin, mcg | 300 | 300 | 300 |
| D-pantothenic acid (calcium D-pantothenate), mg | 10 | 10 | 10 |
| Folic acid, mg | 1 | 1 | 1 |
| Niacinamide, mg | 20 | 20 | 20 |
| Vitamin B1 (thiamine mononitrate), mg | 1.5 | 1.5 | 1.5 |
| Vitamin B12 (cyanocobalamin), mcg | 6 | 6 | 6 |
| Vitamin B2 (riboflavin), mg | 1.7 | 1.7 | 1.7 |
| Vitamin B6 (pyridoxine hydrochloride), mg | 10 | 10 | 10 |
| Vitamin C (ascorbic acid), mg | 100 | 100 | 100 |
|  |  |  |  |

N (%) or median (inter-quartile range) where appropriate.
